# Changes in newspaper coverage of mental illness from 2008 to 2016 in England

**DOI:** 10.1017/S2045796018000720

**Published:** 2018-12-04

**Authors:** C. Anderson, E. J. Robinson, A.-M. Krooupa, C. Henderson

**Affiliations:** 1 Health Service and Population Research Department, Institute of Psychiatry, Psychology and Neuroscience, King's College London, London, UK; 2 Department of Biostatistics & Health Informatics, Institute of Psychiatry, Psychology and Neuroscience, King's College London, London, UK; 3 Marie Curie Palliative Care Research Department, Division of Psychiatry, University College London, London, UK

**Keywords:** Public mental health, stigma, schizophrenia

## Abstract

**Aims:**

Since 2008 England's anti-stigma programme Time to Change has lobbied media outlets about stigmatising coverage and worked with them to promote accurate and non-stigmatising coverage. While this may have an impact on coverage and hence attitudes, it is also possible that coverage can change in response to improving attitudes, through the creation of a market demand for less stigmatising coverage. This study evaluates English newspaper coverage of mental health topics between 2008 and 2016.

**Method:**

Articles covering mental health in 27 newspapers were retrieved using keyword searches on two randomly chosen days each month in 2008–2016, excluding 2012 and 2015 due to restricted resources. Content analysis used a structured coding framework. Univariate logistic regression models were used to estimate the odds of each hypothesised element occurring in 2016 compared with 2008 and Wald tests to assess the overall statistical significance of the year variable as the predictor.

**Results:**

The sample retrieved almost doubled between 2008 (*n* = 882) and 2016 (*n* = 1738). We found a significant increase in the proportion of anti-stigmatising articles (odds ratio (OR) 2.26 (95% confidence interval (CI) 1.86–2.74)) and a significant decrease in stigmatising articles (OR 0.62 (95% CI 0.51–0.75)). Reports on all diagnoses except for schizophrenia were more often anti-stigmatising than stigmatising.

**Conclusions:**

This is the first clear evidence of improvement in coverage since the start of Time to Change. However, coverage of schizophrenia may be less affected by this positive shift than that of other diagnoses. The increase in the level of coverage identified in 2016 requires further investigation, as it may also influence public conceptualisation of what constitutes mental illness, attitudes to mental illness in general and/or specific diagnoses. While most anti-stigma programmes are not diagnosis specific, we suggest their evaluation would benefit from a diagnosis specific approach to allow fuller interpretation of their effects. This could include media analysis driven by hypotheses based on diagnoses to ascertain whether variations by diagnosis over time occur both in the nature and in the proportion of coverage.

## Introduction

Newspaper coverage (the activity of reporting about an event or subject) of mental illness can positively or negatively influence the attitudes of the general public (Philo, 1996; Klin and Lemish, 2008; Corrigan *et al*., 2013). Studies of coverage in many countries show it to be generally inaccurate and stigmatising, as it frequently associates people with mental health problems with violence and criminality, or portrays them as hopeless victims (Coverdale *et al*., 2002). For example, among articles in Italian newspapers related to homicides, suicides and other violent crimes, Carpiniello *et al*. found that those reporting crimes committed by mentally ill people are significantly longer, and contain more pictures and stigmatising language (Carpiniello *et al*., 2007), while Coverdale *et al*. found that in their sample of New Zealand newspaper articles 61.3 and 47.3% referred to dangerousness and criminality respectively (Coverdale *et al*., 2002). Coverage of recovery from and successful treatment of mental health problems has been as little as 4% of mental health articles (Thornicroft *et al*., 2013). Articles discussing psychopharmacological treatments are more critical than articles discussing cardiac medications (Sartorius *et al*., 2010). In addition to contributing to public stigma, negative media representations of mental illness can directly impact on people with mental health problems by reducing their level of self-esteem, discouraging help-seeking behaviours, increasing their experience of discrimination and thus impairing the processes of both personal and clinical recovery (Rusch *et al*., 2005). On the other hand, the media may also be enlisted as a powerful ally in helping to challenge public prejudices, stimulate public debate and project positive, human interest stories about people who live with mental health problems (Klin and Lemish, 2008). For example, promoting news articles portraying depression as a common mental disorder affecting men (as well as women) can challenge stigma (Scholz *et al*., 2014). Recently, national anti-stigma campaigns in Canada (Stuart *et al*., 2014) and New Zealand (Vaughan and Hansen, 2004) have included media professionals as a target group. It was found that, if appropriately enlisted, the media may challenge stigma and disseminate positive mental health messages (Stuart, 2006). Coverage might also improve in response to a positive shift in the attitudes of the population forming the market for a media outlet, to ensure that coverage continues to appeal to consumers. However, the evidence for longitudinal change in reporting is limited (Clement and Foster, 2008; Goulden *et al*., 2011; Murphy *et al*., 2013; Thornicroft *et al*., 2013; Whitley and Berry, 2013*b*; Rhydderch *et al*., 2016). In the context of England's Time to Change anti-stigma programme specifically, there are two reasons to assess media coverage over the course of the programme. First, while change over time cannot be attributed to the programme with complete confidence, assessment for any change in coverage allows for an assessment of the possible effectiveness of this programme's work targeted at media coverage. In Phase 1 (2008–11) of Time to Change, this was largely limited to protesting incidents of particularly stigmatising coverage, for example that which promotes the stereotype of dangerousness. Phase 2 (2011–16), included work with journalists and editors comprising workshops on responsible coverage, and collaboration on development of characters with mental illness portrayed in TV drama series. Second, the overall effect of the mass media on attitudes may moderate the effectiveness of the rest of the programme, which since 2009 has included a targeted social media campaign along with community projects and work with specific groups including youth, employers and medical students. Assessment of changes in coverage over time is therefore useful in interpreting the programme's outcomes with respect to public mental health-related knowledge, attitudes and desire for social distance. These show evidence of improvement since the start of Time to Change (Henderson *et al*., 2016); in the case of attitudes, which have been measured since the mid-1990s, there is evidence of improvement above and beyond the pre-existing trend (Evans-Lacko *et al*., 2014). We previously created a coding framework to assess changes in newspaper coverage over the course of Time to Change (Thornicroft *et al*., 2013). The central theme or idea conveyed in each article was coded into an ‘element’, which was: stigmatising, anti-stigmatising or neutral. These elements were derived from: existing studies of mental health reporting; the wider literature on mental health stigma and inductive coding, in which a sample of articles was qualitatively analysed for recurrent themes and ideas. For example, one stigmatising element identified was ‘hopeless victim’. Vocabulary describing the individual such as ‘consumed by’, and ‘destroyed by’ an illness conveys victim status and weakness. On the other hand, the element ‘risks and causes of mental health problems’ is anti-stigmatising because it reinforces the idea that mental health problems can happen to anyone, and for reasons beyond their control. Each article was then coded overall as stigmatising, anti-stigmatising, mixed or neutral. We found an increase in the proportion of anti-stigmatising articles on mental illness from, 2008–2014 but this was not statistically significant; no reduction in the proportion of stigmatising articles; and fewer articles coded as mixed or neutral (Rhydderch *et al*., 2016).

As 2016 included the end of Time to Change Phase 2 and the start of Phase 3 (2016–21), our aims in this study were to assess the evidence for change over 2008–16 and describe coverage in 2016. We tested the hypotheses that there would be:

1. a significant increase in the overall proportion of anti-stigmatising articles;
2. a significant increase in the proportion of articles featuring the following anti-stigmatising elements:
  - mental health promotion;
  - stigma or
  - injustice;
3. a significant decrease in the overall proportion of stigmatising articles;
4. a significant decrease in the proportion of articles featuring the following stigmatising elements:
  - danger to others or
  - pejorative language;
5. a significant increase in the proportion of sources who are:
  - people with a mental illness;
  - family/friends/carers or
  - mental health charities.

## Methods

The Lexis Nexis Professional UK electronic newspaper database was used to search articles from 27 local and national newspapers which were published on two randomly chosen days each month, and which referred to mental illness.

Ten national mass circulation (>100 000), daily newspapers and the eight highest circulation regional newspapers in England at the start of Time to Change were used. To ensure geographical diversity, only one newspaper per town/city was used. The Sun on Sunday is used from 2011 onwards to replace ‘News of the World’ which went out of print in July 2011.

The following newspapers were included: Daily/Sunday Telegraph, Daily/Sunday Mail, Daily/Sunday Star, Daily/Sunday Express, Daily/Sunday Mirror, Times/Sunday Times, Sun/Sun on Sunday, Guardian/Observer, Independent/Independent on Sunday, Birmingham Evening mail, Eastern Daily Press (Norwich), Evening Chronicle (Newcastle), The Evening Standard, Hull Daily Mail, Leicester Mercury, Liverpool Echo, Manchester Evening News and The Sentinel (Stoke).

### Inclusion criteria

Articles were included if they focused on mental illness, i.e., people with mental illness or mental health services. The search terms consisted of 35 general and diagnostic terms covering the full range of mental disorders. This approach follows Wahl's recommendations (Thornton and Wahl, 1996). The full text of articles in the selected newspapers were searched using the following terms (* = wildcard): ‘mental health OR mental illness OR mentally ill OR mental disorder OR mental patient OR mental problem OR (depression NOT W/1 economic OR great) OR depressed OR depressive OR schizo! OR psychosis OR psychotic OR eating disorder OR anorexi! OR bulimi! OR personality disorder OR dissociative disorder OR anxiety disorder OR anxiety attack OR panic disorder OR panic attack OR obsessive compulsive disorder OR OCD OR post-traumatic stress OR PTSD OR social phobia OR agoraphobi! OR bipolar OR ADHD OR attention deficit OR psychiatr! OR mental hospital OR mental asylum OR mental home OR secure hospital’.

### Exclusion criteria

Non-literal and non-clinical references to mental health were excluded, as well as articles which mentioned mental illness only peripherally. Articles which used a search term: (i) in a context unrelated to anyone's mental health (e.g., ‘the government is schizophrenic about this issue’); (ii) described a personal but non-clinical use (e.g., ‘I'm feeling a bit depressed about this’) or (iii) where diagnostic or slang terms were used metaphorically (e.g., ‘he's driving me nuts’) were excluded. Articles relating primarily to developmental disorders (e.g., autism), neurodegenerative diseases (e.g., Alzheimer's), or alcohol/substance abuse were excluded.

Because of the twofold increase in article numbers between 2008 and 2016 and the increase in workload that would result from coding this sample, in 2016 we introduced random sampling of 50% of the articles from each selected day to create the sample for coding.

### Coding

Articles were coded for their: date; newspaper origin; article type (news, features or opinion); diagnoses mentioned and any person/source directly or indirectly quoted. We used the same coding criteria as for previous work (Thornicroft *et al*., 2013; Rhydderch *et al*., 2016) described in the Introduction, for which detailed codebook was developed outlining the criteria to be used in coding. The central theme or idea conveyed in each article was coded into an ‘element’, which was: stigmatising, anti-stigmatising or neutral. These elements were derived from: (a) existing studies of mental health reporting; (b) the wider literature on mental health stigma and (c) a process of inductive coding, in which a previous sample of articles was qualitatively analysed for recurrent themes and ideas. Elements were classed as primary or secondary depending on where they appeared in the article and in how much of the article they appeared. Finally, each article was coded overall as stigmatising, anti-stigmatising, mixed or neutral. This was based not only on the elements present but the messages conveyed and the overall weight they were given.

The researcher coding the 2016 articles was trained in the same way as those who coded previous years other than the codebook developer (Thornicroft *et al*., 2013; Rhydderch *et al*., 2016). Each researcher coded articles from two days in 2008 which had been coded by the codebook developer. Areas of discrepancy were discussed with CH and then a further two days’ worth of articles previously coded by the codebook developer was coded. Once the *κ* analysis gave a score of over 0.7, indicating substantial agreement, the coder proceeded with individual coding of the 2016 articles. In 2016 the code achieved a *κ* of 0.72, in line with the minimum *κ* score between pairs of coders for previous years of 0.73.

### Analysis

Frequencies and proportions of elements, sources and diagnoses featured in the articles were determined and have been reported; each counted as occurring at least once per article or not at all, i.e., whether present or not. For the sources only, the number of times that each type of source was used in an article was also counted and categorised (0, 1, 2 or 3+ times), for use in the regression analysis.

Univariate logistic regression models were used to estimate the odds that each of the hypothesised elements and each overall category would occur in 2016 compared with the 2008 baseline data. All models were in the following form: dependent variable = element (binary: not occurring (reference) *v*. occurring); and independent variable  =  year (categorical: 2008 (reference), 2009, 2010, 2011, 2013, 2014, 2016). A Wald (*χ*^2^) test was used to assess the overall statistical significance (*α* = 0.05) of the year variable as the predictor in each model. A Bonferroni adjustment was calculated for the *p*-values of the 19 Wald tests (*α*  =  0.05/19  =  0.0026) to aid interpretation and reduce the probability of making a type 1 error (concluding there is a difference when there is none).

To test whether each of the nine sources was more or less likely to be used in 2016 compared with 2008, ordered logistic regression was used: dependent variable  =  source (ordered category: 0, 1, 2 or 3+ times); and independent variable  =  year (as above). The overall significance of the year variable was also tested using a Wald (*χ*^2^) test, and a Bonferroni adjustment was calculated (*α*  =  0.05/9  =  0.0056).

## Results

### The sample

After exclusions, including duplicates and those not meeting the inclusion criteria, a total of 1738 articles were left. This compares to: 941 articles in 2014; 934 in 2013; 698 in 2011; 627 in 2010; 794 in 2009; and 882 in 2008. As our capacity for coding did not extend to this sample size only a 50% sample (*n*  =  869) of these articles was coded, so that a similar number to all previous years was coded.

### Balance of coverage in 2016

Hypotheses (1) a significant increase in the overall proportion of anti-stigmatising articles and (3) a significant decrease in the overall proportion of stigmatising articles.

**Fig. 1.**
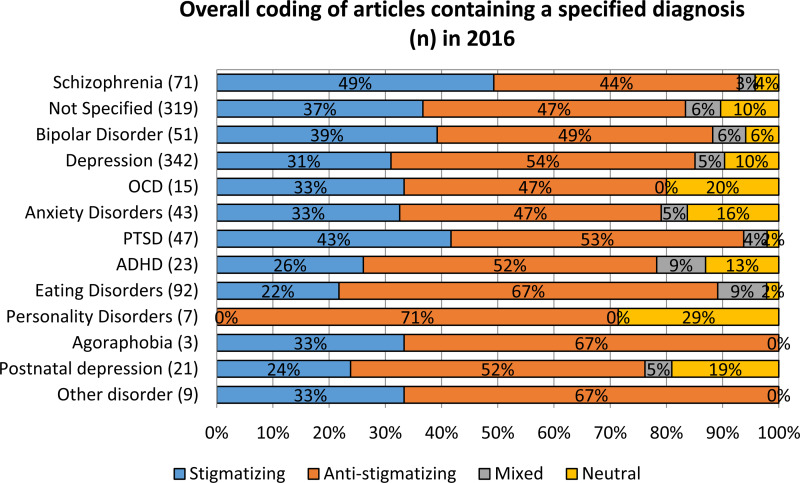
Overall coding of articles containing a specified diagnosis in 2016.

Overall, more articles were anti-stigmatising (50%) than stigmatising (35%) with the remainder mixed (6%) or neutral (9%). However this pattern was not consistent across all diagnoses. Figure 1 shows the number of articles that featured each type of diagnosis in 2016 and the proportion of overall elements for each. Coverage by diagnosis was not the subject of any of our hypotheses, so rather than apply statistical tests we show descriptive statistics to provide context for the results. The positive balance for coverage applied to coverage of all diagnoses except schizophrenia; 48% of articles in which it was featured were coded as stigmatising *v.* 44% stigmatising.

### Changes in elements reported

Hypotheses (2) a significant increase in the proportion of articles featuring the following anti-stigmatising elements: (2a) mental health promotion; (2b) stigma or (2c) injustice; and (4) a significant decrease in the proportion of articles featuring the following stigmatising elements: (4a) danger to others or (4b) pejorative language.

Table 1 shows the numbers and frequencies for coverage elements as a proportion of articles in which the elements appear, and overall categories for each year. The numbers and frequencies as a proportion of the total number of elements more easily reflect the totality of coverage in the sample and are shown in online Supplementary material. Table 2 shows results of univariate logistic regression models estimating the odds that each of the hypothesised elements and each of the overall categories would occur in 2016 compared with the 2008 baseline data, and of Wald (*χ*^2^) tests to assess the overall statistical significance of the year variable as the predictor in each model.

**Table 1.** Frequencies and proportions of elements and overall categorisation across articles, by year

	Year													
Number of articles	2008 (*n* = 882)		2009 (*n* = 794)		2010 (*n* = 630)		2011 (*n* = 698)		2013 (*n* = 934)		2014 (*n* = 941)		2016 (*n* = 869)	
Element	%	*n*	%	*n*	%	*n*	%	*n*	%	*n*	%	*n*	%	*n*
Neutral (overall)	16	145	15	120	11	69	8	57	15	143	14	132	9	82
Mixed (overall)	7	58	6	48	5	30	5	37	6	58	7	67	6	50
Stigmatising (overall)	46	406	43	342	50	316	45	316	38	359	44	411	35	300
Danger to others	21	186	17	138	21	130	14	95	8	74	12	109	17	148
Problem for others	7	62	11	85	9	54	7	50	4	38	7	64	6	54
Hopeless victim	16	137	9	72	13	83	22	153	12	113	29	277	14	123
Strange behaviour	12	108	9	72	9	58	13	93	22	204	16	152	10	88
Personal responsibility	13	114	7	52	3	20	2	11	0	2	8	77	5	40
Sceptical of seriousness	2	18	3	21	1	6	3	19	6	53	3	29	3	26
Pejorative language	6	49	8	61	4	26	4	31	11	106	5	50	4	31
Anti-stigmatising (overall)	31	273	36	284	34	212	41	288	40	373	35	331	50	437
Sympathetic portrayal	23	202	24	193	11	70	20	142	14	127	13	118	31	268
Causes of mental illness	13	117	16	127	11	68	11	79	32	300	9	86	16	139
Recovery from/successful treatment	9	76	7	53	10	60	14	99	19	176	11	100	6	50
Mental health promotion	7	59	5	41	4	26	18	125	9	80	11	108	13	115
Stigma	1	11	2	16	1	7	2	16	6	56	4	34	5	44
Injustice	5	42	7	55	4	25	4	30	13	125	9	88	9	80
Prevalence	3	23	3	25	4	25	4	27	7	62	4	38	4	31

**Table 2.** Univariate analyses comparing elements occurring in articles in 2008–2016

Element	OR (95% CI)	*p*-value	Overall Wald test across 7 years (*χ*^2^(6))	*p*-value	Significance of Wald (*χ*^2^) test after Bonferroni adjustment
0. Neutral	0.52 (0.39–0.70)*	<0.001	508.48	<0.001	Sig.
1. Stigmatising					
1.1 Danger to others	0.77 (0.61–0.98)*	0.036	102.14	<0.001	Sig.
1.2 Problem for others	0.88 (0.61–1.28)	0.493	36.68	<0.001	Sig.
1.3 Hopeless victim	0.90 (0.69–1.17)	0.417	179.72	<0.001	Sig.
1.4 Strange behaviour	0.82 (0.61–1.10)	0.185	71.32	<0.001	Sig.
1.5 Personal responsibility causes	0.33 (0.23–0.48)*	<0.001	119.62	<0.001	Sig.
1.6 Sceptical of seriousness	1.48 (0.81–2.72)	0.206	25.86	<0.001	Sig.
1.7 Pejorative language	0.63 (0.40–1.00)*	0.048	56.21	<0.001	Sig.
2. Anti-stigmatising					
2.1 Sympathetic portrayal	1.50 (1.21–1.86)*	<0.001	146.87	<0.001	Sig.
2.2 Causes of MI	1.24 (0.95–1.62)	0.106	280.85	<0.001	Sig.
2.3 Recovery from MI	0.63 (0.44–0.92)*	0.016	154.17	<0.001	Sig.
2.4 MH promotion	2.13 (1.53–2.96)*	<0.001	117.17	<0.001	Sig.
2.5 Stigma	3.62 (1.84–7.13)*	<0.001	42.06	<0.001	Sig.
2.6 Injustice	2.03 (1.38–2.98)*	<0.001	66.07	<0.001	Sig.
2.7 Prevalence	1.24 (0.71–2.18)	0.445	20.35	0.0024	Sig.
Overall element					
Neutral	0.53 (0.40–0.71)*	<0.001	103.76	<0.001	Sig.
Stigmatising	0.62 (0.51–0.75)*	<0.001	272.64	<0.001	Sig.
Anti-stigmatising	2.26 (1.86–2.74)*	<0.001	88.88	<0.001	Sig.
Mixed	0.87 (0.59–1.28)	0.475	312.24	<0.001	Sig.

*Significant at the *p* < 0.05 level; Sig: significant at the *p* < 0.0026 Bonferroni adjusted level.

There was a significant increase (31–50%: odds ratio (OR) 2.26 (95% confidence interval (CI) 1.86–2.74) *p* < 0.001) comparing 2008 *v.* 2016 for the proportion of anti-stigmatising articles, and year of article was significantly associated with this proportion for the overall sample (*χ*^2^(6)  =  88.9; *p* < 0.001), which gives support to Hypothesis 1.

Comparing 2008 and 2016 there was a significant increase (7–13%) in the proportion of articles containing the element ‘mental health promotion’ (OR 2.13 (95% CI 1.53–2.96) *p* < 0.001), and year of article was significantly associated with this proportion for the overall sample (*χ*^2^(6)  =  117.2; *p* < 0.001), therefore there is support for Hypothesis 2a.

Comparing 2008 with 2016 there was a significant increase (1–5%) in the proportion of articles containing the element ‘stigma’ (OR 3.62 (95% CI 1.84–7.13) *p* < 0.001), and year of article was significantly associated with this proportion for the overall sample (*χ*^2^(6)  =  42.1; *p* < 0.001), giving support to Hypothesis 2b.

Comparing 2008 and 2016 there was a significant increase (5–9%) in the proportion of articles containing the element ‘injustice’ (OR 2.03 (1.38–2.98) *p* < 0.001) and year of article was significantly associated with this proportion for the overall sample (*χ*^2^(6)  =  66.1; *p* < 0.001), giving support to Hypothesis 2c.

There was a significant decrease (46–35%: OR 0.62 (95% CI 0.51–0.75) *p* < 0.001) comparing 2008 *v*. 2016 for the proportion of stigmatising articles, and year of article was significantly associated with the overall sample (*χ*^2^(6)  =  272.6; *p* < 0.001). This provides support to Hypothesis 3.

Regarding the stigmatising element ‘danger to others’, a significantly smaller proportion (21% *v.* 17%) of articles had this element comparing 2008 with 2016 (OR 0.77 (95% CI 0.61–0.98) *p*  =  0.036). Across all the years sampled for the study, year of article was significantly associated with this proportion for the overall sample (*χ*^2^(6)  =  102.1; *p* < 0.001), which shows support for Hypothesis 4a.

Comparing 2008 with 2016 there was a significant decrease (6 to 4%) in the proportion of articles containing the element ‘pejorative language’ (OR 0.63 (95% CI 0.40–1.00) *p*  =  0.048) and year of article was significantly associated with this proportion for the overall sample (*χ*^2^(6)  =  56.2; *p* < 0.001), providing support for Hypothesis 4b.

The Wald (*χ*^2^) tests for all elements and for all categories of overall rating type remained statistically significant after Bonferroni adjustment (*p* < 0.0026). However, multiple testing has not been taken account of for each of the 2008–2016 comparisons, therefore *p*-values close to 0.05 should be interpreted with care.

### Sources of comments and quotations

(5) a significant increase in the proportion of sources who are: (5a) people with a mental illness; (5b) family/friends/carers or (5c) mental health charities.

Table 3 shows the distribution of source types by article (see online Supplementary table for the distribution of sources by the total number of sources found). Table 4 shows results of univariate ordered logistic regression models, estimating the probability that each of the sources would be used in 2016 compared with 2008. There was a significant increase in the proportion of articles using people with mental health problems as a source from 2008 to 2016 (15–27%; *p* < 0.001), and year was found to be significantly associated with this proportion for the sample overall (*χ*^2^(6)  =  73.0; *p* < 0.001), which provides support to Hypothesis 5a. There was no significant difference between 2008 and 2016 in ‘family/friends/carers’ being used as a source (12 to 11%; *p*  =  0.334), contrary to Hypothesis 5b. However, year was a significant predictor overall (*χ*^2^(6)  =  20.1; *p*  =  0.0027) since the proportion of sources from ‘family/friends/carers’ varied over time, dropping particularly low in 2011. There was a significant increase in articles using sources from mental health charities between 2008 and 2016 (2–5%; *p*  =  0.001), but year was not significantly associated with this proportion for the sample overall after Bonferroni adjustment (*χ*^2^(6)  =  14.4; *p*  =  0.026), so there is limited support for Hypothesis 5c, partly due to small numbers.

**Table 3.** Frequencies and proportions of sources across articles, by year

	Year													
Number of articles	2008 (*n* = 882)		2009 (*n* = 794)		2010 (*n* = 630)		2011 (*n* = 698)		2013 (*n* = 934)		2014 (*n* = 941)		2016 (869)	
Source	%	*n*	%	*n*	%	*n*	%	*n*	%	*n*	%	*n*	%	*n*
Mental health charities	2	17	3	23	2	14	3	23	3	31	3	27	5	42
Other organisations	5	44	4	34	2	11	1	7	5	50	7	65	7	60
Other individuals	7	65	6	47	7	43	7	50	18	165	27	253	12	106
Criminal justice	10	87	7	54	10	63	8	54	14	132	19	175	11	99
Politicians	4	34	5	37	2	12	1	10	3	29	5	43	3	23
Cultural work	11	94	14	111	8	48	6	39	2	23	12	109	6	52
People with mental health problems	15	135	13	102	16	100	16	114	21	194	23	217	27	235
Family/friends/carers	12	105	12	97	11	68	7	47	12	114	16	151	11	96
Mental health service provider	14	123	13	104	11	68	7	51	13	117	15	143	12	105

**Table 4.** Univariate analyses comparing sources used in articles in 2008–2016

Source	Coef. (95% CI)	*p*-value	Overall Wald test across 7 years (*χ*^2^(6))	*p*-value	Significance of Wald (*χ*^2^) test after Bonferroni adjustment
1. People with MHPs	0.64 (0.40–0.88)*	<0.001	72.95	<0.001	Sig.
2. MH service providers	−0.27 (−0.56 to 0.01)	0.059	20.42	0.0023	Sig.
3. MH charities	0.95 (0.38–1.52)*	0.001	14.35	0.0260	NS
4. Family/friends/carers	−0.15 (−0.44 to 0.15)	0.334	20.10	0.0027	Sig.
5. Politicians	−0.39 (0.93–0.14)	0.151	19.83	0.0030	Sig.
6. Criminal justice	0.06 (−0.24 to 0.37)	0.681	28.99	<0.001	Sig.
7. Cultural work	−0.63 (−0.98 to −0.28)*	<0.001	98.87	<0.001	Sig.
8. Other organisations	0.28 (−0.12 to 0.69)	0.171	39.53	<0.001	Sig.
9. Other individuals	0.51 (0.18–0.84)*	0.002	176.74	<0.001	Sig.

*Significant at the *p* < 0.05 level; Sig: significant at the *p* < 0.0056 Bonferroni adjusted level; NS: not significant at the *p* < 0.0056 Bonferroni adjusted level.

## Discussion

Over the 9-year period evaluated, the number of articles covering mental health stories in England has significantly increased. This supports previous research findings that mental health coverage in the UK is increasing disproportionately to increases in other news stories (Murphy *et al*., 2013). Our findings suggest that there has been an increase in the proportion of articles which present mental illness in an anti-stigmatising manner and a simultaneous proportional decrease in the depiction of mental illness as stigmatising in newspaper coverage. It thus appears that the increase in coverage observed over time coincides with a shift towards more positive coverage.

Over this time period there have been significant improvements in mental health-related knowledge and attitudes, and a reduction in the desire for social distance as measured by the Attitudes to Mental Illness survey (Henderson *et al*., 2016). Additionally, significantly more respondents in this survey report personal familiarity with mental illness, as a result of personal experience or knowing someone with personal experience. Reported contact with someone with a mental illness has increased particularly among women; this may be related to the rising prevalence of common mental disorder among particularly young women (McManus, 2016). It is possible that a positive feedback loop is in operation; as attitudes improve and the desire for greater understanding of mental health problems increases, newspapers have responded with increasing levels of more positive coverage, which then further influences public attitudes. This was not clearly apparent for the period 2008–14, for which changes were not demonstrated consistently over the years studied (Rhydderch *et al*., 2016). In addition, the improvement in coverage by the end of the second phase of Time to Change supports the change between phases 1 and 2 in the programme's methods of engagement with the media. As described in the Introduction, Phase 2 included guidance on coverage and workshops to promote this guidance, instead of solely protest at stigmatising coverage. This change is supported by the relatively stronger evidence for education as opposed to protest as an anti-stigma strategy (Corrigan *et al*., 2012).

The more stigmatising coverage of schizophrenia is consistent with other UK studies. Two previous studies (Clement and Foster, 2008; Goulden *et al*., 2011) showed little change in coverage related to this diagnosis, while one of these showed that coverage regarding depression had improved (Goulden *et al*., 2011). A study of Scottish newspaper coverage showed a shift in the coverage of schizophrenia such that the emphasis on violence was partially replaced by more subtle forms of stigma over the course of the early years of the See Me anti-stigma programme (Knifton and Quinn, 2008). In several Asian countries the term for schizophrenia has been changed to try to reduce the associated stigma. While the results of a systematic review (Yamaguchi *et al*., 2017) suggested this has had a positive impact on public attitudes, there is less evidence so far for an improvement in media coverage. This pattern of results is similar to that for the evaluations of the first two phases of Time to Change, at the end of which there was evidence for improvement in public stigma-related knowledge and attitudes and reduced desire for social distance (Henderson *et al*., 2016), but little evidence for significant changes in newspaper coverage (Rhydderch *et al*., 2016). Together these studies and the current one suggest that coverage follows rather than leads attitude changes. It is also possible in England that attitudes to schizophrenia lag behind those towards other diagnoses; as the Attitudes to Mental Illness survey is not diagnosis specific, whether this is the case is currently not known.

### Strengths and limitations of the study

As newspaper circulations decline and the use of social media increases, the relative importance of newspaper coverage as an influencer of public attitudes may be falling. Acknowledging this change since 2008, we discuss the other strengths and limitations of this study with reference to Whitley *et al*.’s five domains of difficulty in analysing media representations of mental illness (Whitley and Berry, 2013*a*): (i) *defining relevant search terms:* it is possible that the search terms used did not identify all articles that could convey references to mental health, although pilot searches for non-diagnostic terms such as ‘stress’ and ‘breakdown’, as well as a long list of slang terms, revealed that they yielded no additional, relevant stories. (ii) *Developing appropriate inclusion and exclusion criteria:* we have excluded articles relating to neurodevelopmental and neurodegenerative conditions; conditions such as dementia and autism have been prominent in the media over the last 9 years, and this study may have therefore missed changes in articles related to these conditions. (iii) *Creating a coding scheme:* the coding framework for this study was designed with reference to three sources: existing studies of mental health reporting, the wider literature on mental health stigma, and a process of inductive coding, in which a sample of articles was qualitatively analysed for recurrent themes and ideas. (iv) *Choosing strategies of analysis and dissemination:* this study was designed as a quantitative analysis, in order to facilitate statistical analysis of changing reporting over time. We focused on content analysis of the text in the articles, and did not code other powerful contextual aspects related to the article, such as photographs and headlines used, and placing of the article. (v) *Staffing and training issues:* the newspaper articles were coded by different research workers, although all researchers used the same detailed codebook, and differences in coding were minimised by using trial periods of coding, assessment of agreement levels and discussing discrepancies with other coders.

### Implications for anti-stigma programmes and their evaluation

It may be that newspapers are now more sensitive to changes in market demand as their circulations decline. Thus, if social marketing campaigns directed at the general public have an impact on public attitudes, this may provide a lever to influence press coverage. The other possible mechanism of change in coverage is in direct response to lobbying from and/or work with mental health charities and other organisations. A recent systematic review of evidence for interventions to improve coverage found a limited number of studies of a variety of interventions, including guidelines, education and contact-based education. The results were variable, but the findings of the review, of studies of coverage, and of reviews of anti-stigma interventions more broadly (Mehta *et al*., 2015) provide the current evidence base for anti-stigma programmes considering the mass media as a target group. The evidence for contact-based education in multiple target groups suggest that future interventions could focus on training and empowering people with experience of mental health problems to engage with journalists as sources. This applies especially to individuals with mental health problems which appear to be more often portrayed in a stigmatising manner, in particular schizophrenia. Finally, the time period over which this change in coverage has occurred reinforces the need for long term programmes to reduce stigma and discrimination towards people with experience of mental illness, particularly those whose illness are associated in the media with violence.

A question for further research on media coverage of mental illness is whether, as coverage of mental health problems increases, the proportion of the coverage relating to common mental disorders is increasing. This has important implications for stigma research and the evaluation of stigma reduction interventions. One consequence is that respondents to questions about mental illness may be increasingly thinking of common mental disorder, or even problems such as stress, instead of severe mental illness when they respond. The use of questions about what people conceive of as a mental illness as part of the Time to Change evaluation suggests that the conceptualisation if illness is broadening, with increasing proportions of respondents stating that grief and stress are illnesses in 2017 compared with 2009 (Henderson and Robinson, 2017). This broader conceptualisation might have an influence on attitudes to all mental illness; alternatively, attitudes to common mental disorder may improve due to the increasingly anti-stigmatising coverage of common mental disorder but less so or not at all to severe mental illness. This differential response cannot be detected by attitude scales which use questions about mental illness in general. While most anti-stigma programmes are not diagnosis specific, we suggest their evaluation would benefit from a diagnosis specific approach to allow fuller interpretation of their effects, using attitude measures which ask about diagnosis (Crisp *et al*., 2005) or use vignettes of depression and schizophrenia (Schomerus *et al*., 2012). This could also include media analysis driven by hypotheses based on diagnoses such as that by Goulden *et al*. (2011), to ascertain whether variations by diagnosis over time occur both in the nature and in the proportion of coverage. Finally, a wider scope of media for analysis and attention to visual media would allow a fuller assessment of the media's influence on attitudes to mental illnesses.

## Availability of data and materials

The dataset of newspaper articles and the coding dataset are available upon reasonable request.
